# Long-Time Relaxation of Stress-Induced Birefringence of Microcrystalline Alkali Halide Crystals

**DOI:** 10.3390/molecules23040757

**Published:** 2018-03-25

**Authors:** Hiroki Ueno, Ryoga Arakane, Yoshihisa Matsumoto, Tomoki Tsumura, Akihito Kitazaki, Toru Takahashi, Shotaro Hirao, Yasushi Ohga, Takunori Harada

**Affiliations:** 1 Department of Integrated Science and Technology, Faculty of Science and Technology, Oita University, Dannoharu, 700, Oita city 870-1192, Japan; v16e4005@oita-u.ac.jp (H.U.); ttsumura@oita-u.ac.jp (T.T.); v17f1001@oita-u.ac.jp (A.K.); ttakaha@oita-u.ac.jp (T.T.); hirao-shoutarou@oita-u.ac.jp (S.H.); yohga@oita-u.ac.jp (Y.O.); 2 Department of Mechanical Engineering, National Institute of Technology, Oita College, Maki, 1666, Oita city 870-0152, Japan; ryouga110714@gmail.com (R.A.); matumoto@oita-ct.ac.jp (Y.M.)

**Keywords:** alkali halides, tablet method, birefringence, chiroptical measurement

## Abstract

Alkali halide single crystals are most commonly used as the diluent matrix in the tablet method or disk technique for spectroscopic measurements. However, stress-induced birefringence (SIB) of alkali halides as well as intrinsic birefringence manifest during the disk formation process. Thus, the true chiroptical measurement is disturbed by optical anisotropies (OA) containing SIB and intrinsic birefringence, except in the case of optical homogeneity. SIB is generally larger than intrinsic birefringence and has a value of several thousand millidegrees in the ultraviolet-visible wavelength range, although this varies with disk type. Here, to investigate the SIB origin, alkali halide crystals were examined using polarized light, X-ray diffraction, Fourier-transform infrared, and electron backscattering diffraction spectroscopic measurements. It was found that, after stress release, the SIB exhibited nonlinear long-time relaxation, which roughly converged within several hours, with the only time-invariant intrinsic birefringence remaining being due to OA. This behavior was strongly related to an increase in the quasi-amorphous domain and the generation of an air gap between the crystallite boundaries and their pellets. Further, a straightforward correlation was found between amorphization and an increase in the disk water content caused by deliquescence. Thus, the OA of alkali halide single crystals was found to have two different origins yielding intrinsic birefringence and SIB.

## 1. Introduction

Solid-state chiroptical spectroscopy has begun to attract considerable attention as a new analytical tool for many research fields involving solid-state chirality. Absorption, emission, diffuse reflection, and scattering techniques, which are based on circular dichroism (CD) (electronic (ECD) [1] and vibrational (VCD) [2]), circularly polarized luminescence [3], diffuse reflectance CD [4], and Raman optical activity (ROA) [5,6], respectively, can be used with solid-state samples with care.

The tablet or matrix-disk method is one of the most popular sample preparation techniques for a wide range of spectroscopic measurements for solid samples, except in cases to which the single-crystal method is applicable. The representative diluent matrix for the tablet method or disk technique is a cubic alkali halide, e.g., KBr, KCl, or KI. These cubic alkali halides are transparent in the ultraviolet-visible (UV-Vis) to infrared wavelength ranges (ca. 0.2–20 μm). However, there is a tacit understanding that stress-induced birefringence (SIB) of alkali halides occurs during the disk formation process, the nonlinear long-term relaxation of which is empirically known. The optical anisotropy (OA) consists of both intrinsic birefringence belonging to a symmetry break related to the wave vector **q**-dependence of the dielectric constant [7,8,9,10] and an extrinsic one (SIB) induced by the pressure in the disk formation. SIB is large enough compared to the intrinsic birefringence. The OA associated with the SIB generally has values exceeding 2000 mdeg/mm (1 mdeg = 3.05 × 10^−5^ optical density (OD)) in the UV-Vis region, corresponding to the electronic transitions of most organic compounds, and is not negligibly small for a relatively weak chiral signal (~20 mdeg). Thus, careful polarization analysis is indispensable for true chiroptical measurements of chiral samples dispersed in an optically anisotropic matrix. Such measurements are performed using modern chiroptical spectrophotometers such as ECD and circularly polarized luminescence spectrophotometers. These devices are based on polarization-modulation techniques, because it is necessary to couple non-ideal optics and electronics with strong OA, i.e., with nonchiral signals related to linearly polarized phenomena [1,11]. However, thus far, no detailed analysis of OA has been conducted, and no method for minimizing this phenomenon has been reported.

In this paper, we thoroughly investigate the (intrinsic + extrinsic) OA mechanism inducing nonlinear SIB relaxation in the alkali halide disks used in matrix-disk methods, and we present an essential matrix-disk method for analyzing chiral signals for cases in which a dedicated solid-state chiroptical instrument is unavailable. The potential influence of artifact signals, originating from the coupling of instruments with non-ideal optical properties, on the true chirality signal should be investigated. When the OA after the relaxation of SIB is negligibly small compared with the net chirality signal, an artifact-free chiral signal can be obtained in the polarization-modulation spectroscopic measurements.

## 2. Experimental

KBr, Cl^−^, and I^−^ single crystals were purchased from JASCO Co., Ltd. (Tokyo, Japan), Wako Chemical Co., Ltd. (Tokyo, Japan), and Tokyo Chemical Industry Co., Ltd. (Tokyo, Japan), respectively. Crystals of dry KBr, KCl, and KI (130 mg) were finely ground and the powder was compressed at 14–70 MPa/cm^2^ in vacuum for 15 min to prepare a transparent disk with a diameter of 1 cm and a thickness of 0.7 mm. A KBr single crystal (space group: *F*m-3m), which was cleaved using a surgical blade to make plates of 0.6, 0.8, and 0.9 mm thickness perpendicular to the (100) face, was hydrostatically compressed at 100 MPa/cm^2^ in a hexane solution using a high-pressure cell. The Miller indices of the KBr cleaved faces were investigated using an X-ray diffractometer (Bruker APEX CCD (Madison, WI, USA)). We used a comprehensive chiroptical spectrophotometer (CCS: J-700CPL (JASCO, Tokyo, Japan)), which can measure all polarization phenomena, i.e., CD, circular birefringence (CB), linear dichroism (LD), linear birefringence (LB), and circularly polarized luminescence (CPL) [11]. The details of the CCS have been described previously [11]. The KBr disk or KBr single crystal, held by a specially designed sample rotation holder, was placed normal to a light beam. LB measurements were performed by a CCS equipped with a Glan-Taylor prism (optical axis at 45° with respect to the *y*-axis) placed in front of the sample (Supplementary Materials). The wavelength-scan LB spectrum was recorded over a wavelength range of 700–230 nm with ‘’standard’’ sensitivity at 100 nm/min, with a 1-nm resolution and a time constant of 1 s. The rotational LB spectra were obtained through computer-controlled rotation of the sample by 360° in the (*X*-*Y*) plane at a speed of 2.5°/s, at 300 nm, for disks and single crystals. The Stokes-Mueller matrix method was used for the data analysis. This process was repeated for KCl and KI disks and single crystals.

Powder X-ray diffraction (XRD) measurements were performed with a Rigaku RINT-Ultima III diffractometer (Tokyo, Japan) at a scanning rate of 0.25°/min in the 2θ range from 10°–80° using graphite-monochromated Cu-Kα radiation (λ = 0.154059 nm). Field emission scanning electron microscopy (FE-SEM) images were taken using an electron microscope (JEOL JSM-7100F FE-SEM (Tokyo, Japan)). Specimen microstructures were characterized using a JEOL JSM-7100F FE-SEM equipped with an electron backscattering diffraction (EBSD) detector (HKLNordlys, Oxford Instruments, Oxon, UK). The EBSD data were acquired using Flamenco software, included in the Channel 5 software or the AZtec software, both from Oxford Instruments (Oxon, UK). The SEM and EBSD settings were selected to achieve the optimal spatial resolution and signal strengths for measurements of heavily deformed microstructures. To eliminate electron charging samples with poor electrical conductivity, those samples were coated with a thin conductive film before being placed in a high-vacuum SEM chamber (high vacuum: ~10^−5^ Pa). The disks were prepared using optimal carbon coat thicknesses, i.e., 3–5 nm, which are generally used for EBSD [12]. The KBr disks were characterized based on their grain size, orientation, and misorientation by an EBSD system attached to the FE-SEM. Fourier-transform infrared (FT-IR) spectroscopy was performed using an FT/IR-470Plus (JASCO, Tokyo, Japan) at a relative humidity range of 20–50%.

## 3. Results

**Figure 1:**
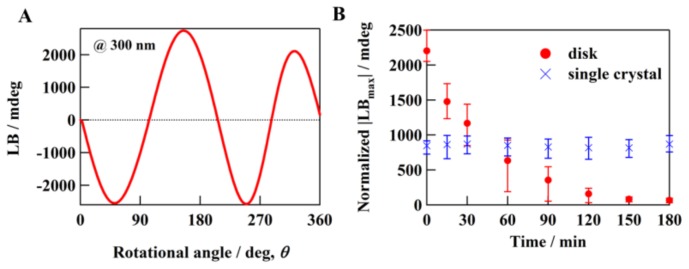
(**A**) Linear birefringence (LB) rotational measurement of KBr disk at 300 nm; (**B**) Recovery process of stress-induced LB values (@ 300 nm) of KBr disk (circle) and single crystal (x) at plus LB_max_ positions; LB values normalized with respect to sample thickness.

Ten disks with identical thicknesses (0.7 mm) for a KBr, KCl, or KI matrix were studied. Figure 1A shows a representative (typical) LB rotational measurement result for a 50-kHz signal at 300 nm, which was obtained by rotating a KBr disk mounted on the sample rotation holder. Note that a 50-kHz signal obtained with a polarizer can generally be regarded as an LB signal, because the single-order term, LB, is much larger than the second-order term, LD, as shown in Equation (S14) (Supplementary Materials). As expected, when a light beam was incident on the freshly prepared KBr disk, being exactly normal to the wide face, a strong LB signal with sin2θ periodicity appeared in the LB rotational measurements (Figure 1A). This result is evidence that the KBr disk has extrinsic or intrinsic OA. In contrast, when the light beam was incident on a KBr crystal pressed at 100 MPa/cm^2^ in a hexane solution, exactly normal to the (100) face, a weak LB signal was detected in the LB rotational measurements. The shape was a trigonometric function (data not shown). Thus, it is clear that cubic KBr single crystals exhibit intrinsic birefringence even though these crystals constitute an isometric system. The birefringence of KBr disks has stronger angular dependence than that of single crystals, which is in agreement with experimental results that cubic single crystals possess intrinsic birefringence even though they belong to an isometric system [10].

Time-course LB signals (@ 300 nm) for both the KBr disks and single crystals were measured at the LB maximum position, LB_max_, obtained via the LB rotational measurements (Figure 1B). Nonlinear OA relaxations were only observed for the KBr disks, having relaxation times of several hours, although the LB intensities of the disks were uneven. Thus, the results suggest that the LB signals contain extrinsic OA signals; this is explained in the discussion section below. Further, the decay curve does not pass through zero, which may be due to the intrinsic OA. Note that the measured LB values and averaged intrinsic OA values of the crystallites in the disk do not necessarily correspond to the single-crystal values.

The relationship between the LB intensities and pressure on the disk was quantitatively analyzed. Figure S2 presents plots of the LB_max_ values for selected disks (300–2500 mdeg) as functions of pressure (14–70 MPa/cm^2^). No relationship between pressure and LB_max_ was identified. To achieve a spectroscopically measurable transparent disk, a pressure of more than 55 MPa/cm^2^ is required. Therefore, in all experiments, the pressure for disk formation was set to 70 MPa/cm^2^.

We further conducted LB measurements for single crystals and disks of cubic KCl and KI crystals (space group: *F*m-3m) using the CCS. These measurements were identical to those performed for the KBr specimens described above. As expected, these single crystals also exhibited intrinsic birefringence similar to the KBr single crystal. A nonlinear OA relaxation that converged within several hours was observed, in agreement with the results for the KBr disks. Note that the convergence time of the nonlinear OA relaxation is inversely proportional to humidity, and accelerates in the order of KI, KCl, KBr. The thermal variation of the refractive index was also investigated. The change in temperature, Δ*T*, for alkali halides in the process of disk formation and time-course measurements was found to be very small, at Δ*T* <≈ 2 °C. We found the value of the refractive index for KBr to be 1.556 ± 0.001 in this range. These results indicate that the observed OA (=LB) was the summation of the SIB originating from the external influence of distortion and the intrinsic LB. Further, immediately after stress release, the SIB of the alkali halides exhibited a nonlinear long-time relaxation, which roughly converged within several hours. Only intrinsic birefringence remained as the OA.

**Figure 2:**
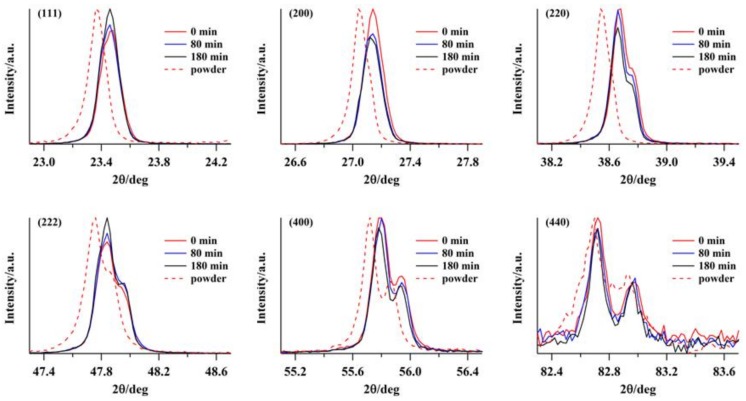
Time-dependent variations in X-ray diffraction (XRD) patterns of alkali halide KBr disk and KBr microcrystalline powder (dotted lines), with several peaks, i.e., (111), (200), (220), (222), (400), and (440), under exposure to air (40-min intervals). XRD patterns (0, 80, and 180 min) selected from interval data are indicated.

Figure 2 shows the time-dependent variations in the XRD patterns of the alkali halide KBr disk at several representative diffraction peaks, i.e., (111), (200), (220), (222), (400), and (440), under exposure to air. This behavior was monitored in 40-min intervals from the initial state (0 min), which occurred immediately after pressure release. For all diffraction peaks, small changes in these intensities and the full width at half maximum (FWHM) were observed with time. The FWHM was dependent on the particle size (coherent scattering regions) and strain induced in the individual crystal lattice. The FWHMs gradually became ~10–20% narrower than those of the initial state, and diffraction peak shifts from the initial values to higher diffraction angles were concomitantly observed. Thus, these results indicate that the disk formation processes alter the cubic crystal lattice to another form. Immediately after pressure release, however, the interatomic distance begins to gradually shorten, ultimately returning to the original cubic distance. The correlation of the intensity changes with the long-term relaxation of the SIB was also obtained. It was found that, when the intensities of the (111) and (222) diffraction peaks decrease, those for the (200) and (400) peaks increase in contrast, and vice versa. Thus, a crystallite reorientation within the disk structure occurs, which may be attributed to the quasi-amorphous transformation in the crystallite boundaries, as shown below. Thus, the XRD analysis supports the hypothesis that the observed nonlinear relaxation of the SIB originates from the extrinsic OA.

**Figure 3:**
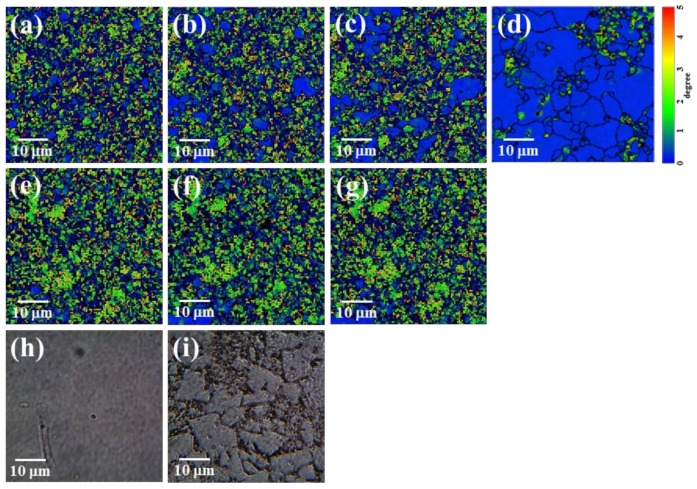
Time-course local misorientation of crystalline KBr in disk measured by electron backscattering diffraction (EBSD) for open-air storage: (**a**) 20 min; (**b**) 50 min; (**c**) 120 min; (**d**) 13 h; and for vacuum storage: (**e**) 30 min; (**f**) 80 min; (**g**) 180 min. Optical microscope images of a KBr disk for the open-air storage case: (**h**) 0 min; (**i**) 180 min. The EBSD measurements were conducted in a high-vacuum SEM chamber.

To investigate the local strain in the crystallite, we conducted FE-SEM and EBSD measurements. From the FE-SEM observations, the grain size was estimated to range from several micrometers to 20 μm, with an average value of 10 μm. EBSD allows measurement of crystal orientations on the specimen surface with a spatial resolution of nanoscale order [13]. The magnitude of the local strain can be estimated from the change in crystal orientation (misorientation) and the misorientation distributions obtained from the EBSD analysis. Figure 3 shows the time-course local misorientations of crystalline KBr in two disks preserved under atmosphere (open-air storage, Figure 3a–d) and vacuum conditions (~10^−5^ Pa, Figure 3e–g), respectively. Here, the initial stage, 0 min, is defined as occurring immediately after KBr disk formation. The colors indicate the misorientation in the range of 0° to 5°, shown in Figure 3 as progressing from blue to red with increasing value. The distribution of the strain in the crystallite was identified using the image correlation technique [14].

The obtained misorientation maps show that the misorientation was distributed evenly over the entire crystallite. For the open-air storage case (Figure 3a–d), it is apparent that the induced local strain in the crystallite gradually released with time from the initial stage to 120 min, before finally evanescing (13 h). Concomitantly, in the crystallite boundaries, the pseudo-amorphous domains (shown in black) inversely increased with time. This transformation from a cubic crystal lattice to a pseudo-amorphous phase may have occurred because of the implanted water molecules on the grain boundary. Thus, enlargement of the pseudo-amorphous region may be strongly associated with dissolution of the local strain in the crystallite. Note that the misorientation dissolution rate was slower than the average LB relaxation time, because a carbon-coated disk was used in the EBSD measurement. On the other hand, for the vacuum storage case, the misorientation exhibited almost no changes until 180 min. These results obviously indicate that there is a difference between the local misorientation behaviors for open-air and vacuum storage. It is speculated that the strain relaxation is directly influenced by the first effect, e.g., pseudo-amorphous transformation related to the water molecules in the open system, and the secondary effect, e.g., generation of the air layer between the crystallite boundaries. Under both conditions, the transformation from transparent to opaque occurs because of the change in the refractive index, which is caused by the pseudo-amorphous transformation and/or generation of an air layer at the crystallite boundaries. Thus, the refractive index, *n*_amor_, of the solid amorphous phase has a small value compared with the corresponding crystalline phase, because the density of the former is lower than that of the latter [15]. In other words, the reflection occurs at an interface of different refractive indices, as indicated by the following observation.

Under optical microscope observation, each crystallite in the final state could be clearly discriminated (Figure 3i); however, discrimination was not possible for specimens in the initial state, as shown in Figure 3h. These findings are also consistent with the decrease in reflectance independently measured by a UV-Vis spectrophotometer (V-770 equipped with an ARMN-920 attachment) (Figure S3). It is speculated that amorphization and air-gap generation induce the change in the refractive index at the crystallite boundaries, resulting in the deterioration of the spectral transmittance and the long-term relaxation of the SIB introduced by the disk formation and consequently connected to the local strain dissolution in each crystallite. In this work, we found a good correlation between the long-time relaxation of the SIB and the increase in the number of water molecules on the disk, as shown below.

**Figure 4:**
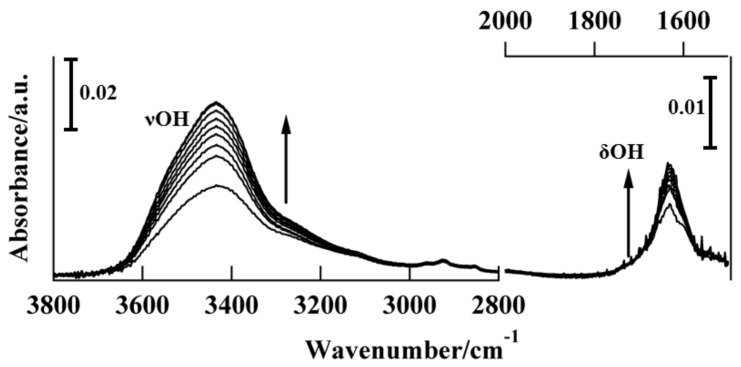
Time-course variations of absorption FT-IR spectra for water adsorption on a KBr disk (15-min intervals). The arrows indicate the direction of the spectral intensity evolution with time.

**Figure 5:**
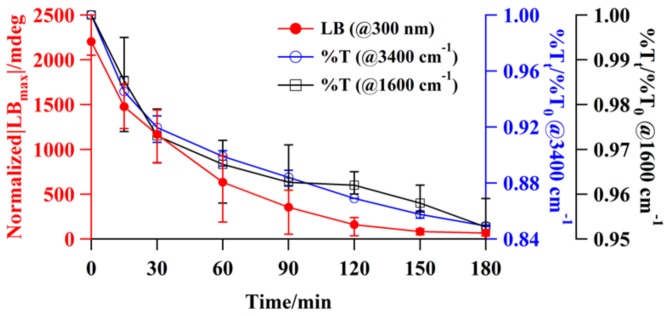
Recovery process of stress-induced LB values (@ 300 nm) of a KBr disk at plus LB_max_ positions, transmission at νOH stretching (3400 cm^−1^), and δOH bending mode bands (1600 cm^−1^), all plotted as functions of time to indicate the adsorption events of the water molecules on the disk. %T_t_ and %T_0_ are FT-IR transmittance at the intermediate and initial stages at the relaxation process, respectively. The data points indicate the mean values (*n* = 10) with standard deviation bars.

Transmission FT-IR spectroscopy was used to investigate the adsorption of water on the alkali halide surfaces, for both single-crystal and particle surfaces. The water uptake on the alkali halide disk as a function of time at room temperature was studied. Figure 4 shows the time-course changes in the absorption FT-IR spectra for water adsorption on the KBr disk. The spectra indicate two broad absorptions of approximately equal intensity with frequencies of νOH (3400 cm^−1^) and δOH (1600 cm^−1^), corresponding to OH stretching vibrations and OH bending modes, respectively [16,17]. Immediately after disk formation, the amplitudes of the fundamental 3400 cm^−1^ νOH stretching band and 1600-cm^−1^ δOH band gradually increased with time, indicating water adsorption on the KBr matrix. The IR absorption intensities related to the water molecules were determined after baseline subtraction based on the peak heights for the 3400 cm^−1^ νOH stretching band. For liquid-like molecular water with a broad band at 3400 cm^−1^, a molar absorption coefficient *ε* [18] of liquid water (8.1 Lmol^−1^mm^−1^) is generally used for the quantitative calculation of the water content of minerals [19,20]. If this value is employed for the 3400 cm^−1^ band of KBr, we find that the water content *c* is ~10^−2^ mol H_2_O/L [21]. Considering the density of KBr (2.75 g cm^−3^), this corresponds to a water fraction change from approximately 1.6 × 10^−2^ (180 min) to 0.58 × 10^−2^ wt % (0 min), i.e., from 157 to 58 ppm. For KCl and KI, the water fraction changed from 131 (180 min) to 63 (0 min) ppm and from 334 (180 min) to 58 (0 min) ppm, respectively. As shown in Figure 5, the decay profile for transmission at the νOH band plotted as a function of time is well correlated with the long-time relaxation of SIB (Figure 1b). However, no facet selectivity of water molecules adsorbed on the KBr surface was observed, as indicated by the lack of angular dependence of the XRD patterns (Figure 2).

These findings indicate that the deliquescence is strongly related to the quasi-amorphous transformation from the solid crystalline phase, because the increase in amorphous domains at the grain boundaries and the long-term relaxation of the SIB are connected to increases in the peak intensities at 3400 cm^−1^ and 1600 cm^−1^, respectively. Based on these results, it is speculated that the quasi-amorphous transformation in the crystallite boundaries influences the distortion elimination in the crystallite, inducing the long-time relaxation of the SIB. To investigate the efflorescence process, alkali halide disks containing water molecules were kept in an oven heated to 200 °C and annealed for 2 h; then, they were gradually cooled to room temperature. Once imbedded in the disk, the water molecules were only minimally removed by the oven treatment. Repeated hydration/dehydration cycling was not exhibited, even for a prolonged heating time; i.e., no efflorescence was observed. In the initial state immediately after disk formation, KBr disks comprised of micron- and submicron-size solid crystalline phase were present in solid-phase form. Under atmosphere, hydration gradually progressed and the solid crystalline phase changed to an amorphous form. These observations indicate that, after the micron- and submicron-size alkali halide particles were deliquesced in this study, the retransformation of the solid crystalline phase from the amorphous solid phase induced by hydration did not occur during the heat treatment. The specimens typically remained in pseudo-amorphous form even under dry conditions [22].

## 4. Conclusions

We proved experimentally that disks made from cubic crystals of point group *O*_h_, such as KBr and KCl, have extrinsic OA. To investigate the origin of SIB in these materials, the crystallite strain and deliquescence were investigated using polarized light, XRD, FT-IR, and EBSD spectroscopic measurements. The long-time relaxation of the SIB induced by disk formation was observed for the alkali halide disks, but not for single crystals. Thus, it was experimentally proven that the SIB is induced by the pressure employed for disk formation, which is directly related to the strain in the crystallite and its deliquescence [23]. The diffraction peaks shifted to higher angles after disk formation, but gradually returned to the original cubic angles with time. In the initial state immediately after disk formation, SIB in each crystallite and grain boundaries in each disk were observed. The SIB decreased with time and converged to a constant value (Figure 1). In addition, the νOH and δOH signals for the water molecules were found to increase with time immediately after disk formation; this finding is well correlated with the long-time relaxation of the SIB. These results strongly suggest that the dominant factor of the SIB (strain around the grain boundary) is extrinsic and not intrinsic (crystallite strain). Moreover, it is speculated that the strain relaxation phenomenon in the crystallite is due to amorphous transformation strongly related to the water molecules.

The physicochemical properties of the examined materials determined by the matrix, i.e., the transmittance, refractive index, and dielectric constant, significantly influence the signals obtained in the high-energy wavelength region. This is because severe artifact signals arise from macroscopic anisotropies that are unique to solid-state samples in the polarization-modulation method. By performing precise polarization-modulation spectroscopic measurements, we obtained detailed information on these optical phenomena as well as the interactions between them and the dilute sample. Here, we must emphasize that true chirality measurement is achieved only through the use of analytical methods [1,3,11] devised to eliminate contributions from macroscopic anisotropies such as nonchiral signals, LB, LD, and linearly polarized luminescence, except in cases where the OA after relaxation of strain originated from the SIB is negligibly small compared with the net chirality signal. The artifact signals originating from the coupling of instruments with non-ideal optical properties must be checked, and the potential influence of these signals on the true chirality signal should be investigated. In general, when the amplitudes of the nonchiral signals are 10 to 100 times larger than that of the chirality signal (~10^−4^ OD), the artifact signal originating from the coupling of non-ideal components (~10^−3^ OD) is of the order of 10^−5^–10^−6^ OD [1,3,11]. In this case, special operation is unnecessary, because the artifact is sufficiently small compared to the true signal. On the other hand, when the nonchiral signals have amplitudes more than 100 times larger than the chirality signal, the artifact signal originating from the coupling effect is not negligibly small. Thus, we believe that the true chirality signal must be obtained using the above polarization analysis method when the artifact signals are larger than the true chirality signal.

## Supplementary Materials

The following are available online: three figures, theoretical Equations (S1–S14). Figure S1: Block diagram of the Comprehensive Chiroptical Spectrophotometer (CCS: J-700CPL), Figure S2: The absolute LB signals of KBr disks made from different pressure, Figure S3: Time-course change of reflection spectra of KBr disk, Theoretical equations (S1–S14).

## References

[1] Kuroda R., Harada T., Shindo Y. (2001). A solid-state dedicated circular dichroism spectrophotometer: Development and application. Rev. Sci. Instrum..

[2] Nafie L.A. (2011). Vibrational Optical Activity: Principles and Applications.

[3] Harada T., Moriyama H., Kuroda R. (2012). Solid-state circularly polarized luminescence measurements: Theoretical analysis. Chem. Phys. Lett..

[4] Bilotti I., Biscarini P., Ferranti F., Castiglioni E., Kuroda R. (2002). Reflectance circular dichroism of solid-state chiral coordination compounds. Chirality.

[5] Barron L.D., Bogaard M.P., Buckingham A.D. (1973). Raman scattering of circularly polarized light by optically active molecules. J. Am. Chem. Soc..

[6] Barron L.D., Bogaard M.P., Buckingham A.D. (1973). Differential Raman scattering of right and left circularly polarized light by asymmetric molecules. Nature.

[7] Agranovich V.M., Ginzburg V.L. (1984). Crystal Optics with Spatial Dispersion, and Excitons.

[8] Burnett J.H., Levine Z.H., Shirley E.L. (2001). Intrinsic birefringence in calcium fluoride and barium fluoride. Phys. Rev. B.

[9] Zaldo C., López C., Meseguer F. (1986). Natural birefringence in alkali halide single crystals. Phys. Rev. B.

[10] Harada T., Sato T., Kuroda R. (2005). Intrinsic birefringence of a chiral sodium chlorate crystal: Is cubic crystal truly optically neutral?. Chem. Phys. Lett..

[11] Harada T., Hayakawa H., Watanabe M., Takamoto M. (2016). A solid-state dedicated circularly polarized luminescence spectrophotometer: Development and application. Rev. Sci. Instrum..

[12] Bestmann M., Pennacchioni G., Frank G., Göken M., De Wall H. (2011). Pseudotachylyte in muscovite-bearing quartzite: Coseismic friction-induced melting and plastic deformation of quartz. J. Struct. Geol..

[13] Pennock G.M., Drury M.R., Peach C.J., Spiers C.J. (2006). The influence of water on deformation microstructures and textures in synthetic NaCl measured using EBSD. J. Struct. Geol..

[14] Oxford Instruments Co. Ltd. (2017). EBSD Technical Note.

[15] Rai R., Ghosh N., Singh B.K. (2015). Effect of humid air exposure on photoemissive and structural properties of KBr thin film photocathode. Nucl. Instrum. Methods Phys. Res. A.

[16] Davis K.M., Agarwal A., Tomozawa M., Hirao K. (1996). Quantitative infrared spectroscopic measurement of hydroxyl concentrations in silica glass. J. Non-Cryst. Solids.

[17] Al-abadleh H.A., Grassian V.H. (2003). FT-IR study of water adsorption on aluminum oxide surfaces. Langmuir.

[18] Thompson W.K. (1965). Infra-red spectroscopic studies of aqueous systems. Part 1.—Molar extinction coefficients of water, deuterium oxide, deuterium hydrogen oxide, aqueous sodium chloride and carbon disulphide. Trans. Faraday Soc..

[19] Aines R.D., Rossman G.R. (1984). Water in minerals? A peak in the infrared. J. Geophys. Res..

[20] Rossman G.R. (1988). Vibrational spectroscopy of hydrous components. Rev. Mineral..

[21] Nakashima S., Matayoshi H., Yuko T., Michibayashi K., Masuda T., Kuroki N., Yamagishi H., Ito Y., Nakamura A. (1995). Infrared microspectroscopy analysis of water distribution in deformed and metamorphosed rocks. Tectonophysics.

[22] Liu Y., Yang Z., Desyaterik Y., Gassman P.L., Wang H., Laskin A. (2008). Hygroscopic behavior of substrate-deposited particles studied by micro-FT-IR spectroscopy and complementary methods of particle analysis. Anal. Chem..

[23] Arima K., Jiang P., Lin D.-S., Verdaguer A., Salmeron M. (2009). Ion segregation and deliquescence of alkali halide nanocrystals on SiO_2_. J. Phys. Chem. A.

