# Supplementary material for: Long-Time Relaxation of Stress-Induced Birefringence of Microcrystalline Alkali Halide Crystals
Source: Molecules. 2018 Mar 25;23(4):757. doi: 10.3390/molecules23040757 (PMC6017667; doi:10.3390/molecules23040757)
Supplement: Supplementary file 1 [file molecules-23-00757-s001.pdf]

Supporting Information

Long-Time Relaxation of Stress-Induced Birefringence of Microcrystalline Alkali Halide Crystals

Hiroki Ueno [1], Ryoga Arakane [2], Yoshihisa Matsumoto [2], Tomoki Tsumura [1], Akihito Kitazaki [1], Toru Takahashi [1], Shotaro Hirao [1], Yasushi Ohga [1] and Takunori Harada [1,*]

[1] Department of Integrated Science and Technology, Faculty of Science and Technology, Oita University, Dannoharu, 700, Oita city 870-1192, Japan; v16e4005@oita-u.ac.jp (H.U.); ttsumura@oita-u.ac.jp (T.T.); v17f1001@oita-u.ac.jp (A.K.); ttakaha@oita-u.ac.jp (T.T.); hirao-shoutarou@oita-u.ac.jp (S.H.);
yohga@oita-u.ac.jp (Y.O.)

[2] Department of Mechanical Engineering, National Institute of Technology, Oita College, Maki, 1666, Oita city 870-0152, Japan; ryouga110714@gmail.com (R.A.); matumoto@oita-ct.ac.jp (Y.M.)

* Correspondence: tharada@oita-u.ac.jp; Tel.: +81-(0)97-554-7622

Theoretical analysis

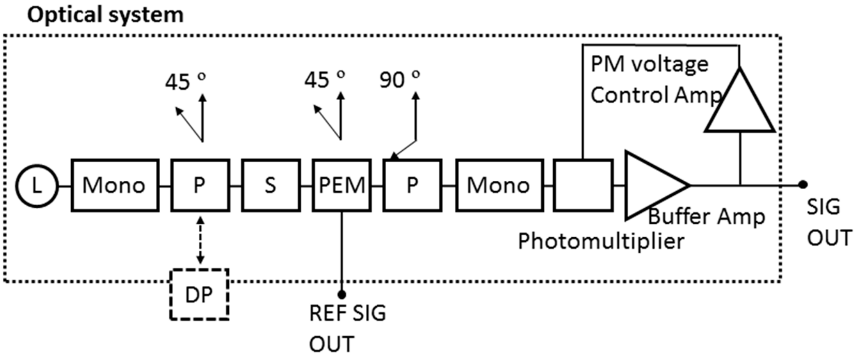

Figure S1. Block diagram of the Comprehensive Chiroptical Spectrophotometer (CCS: J-700CPL): L: light source, Mono: first and second monochromators, DP: depolarizer, P: Polarizers, S: sample, PEM: photoelastic modulator, PM: Photomultiplier.

The light intensity at the detector (PM) can be calculated from the matrix product of $\mathbf{D}\cdot\ \mathbf{P}_{(90)}\cdot\mathbf{M}_{(45,\delta)}\cdot\mathbf{S}_{(\theta)}\cdot\ \mathbf{P}_{(45)}\cdot\ \mathbf{Mo}\cdot\ \mathbf{I}_0$. Here, $\mathbf{D}$, $\mathbf{P}_{(90)}$, $\mathbf{S}_{(\theta)}$, $\mathbf{M}_{(45,\delta)}$, $\mathbf{P}_{(45)}$, $\mathbf{Mo}$ are the Mueller matrix expressions for detector, analyzer, sample, photoelastic modulator (PEM), polarizer and monochromator, respectively. Using the Mueller matrix calculation of $\mathbf{M}_{(45,\delta)}\cdot\ \mathbf{P}_{(45)}\ \cdot\mathbf{Mo}\cdot\ \mathbf{I}_0$, the Stokes vector (1 0 0 0) of the emerging light from the modulator can be expressed as

$$\mathbf{M}_{(45,\delta)}\ \cdot\mathbf{S}_{(\theta)}\cdot\ \mathbf{P}_{(45)}\ \cdot\ \mathbf{I}_0 =$$

$$\begin{pmatrix} \mathbf{1} & \mathbf{0} & \mathbf{0} & \mathbf{0} \\ \mathbf{0} & \mathbf{1} & \mathbf{0} & \mathbf{0} \\ \mathbf{0} & \mathbf{0} & \boldsymbol{cos(\delta+\alpha)} & \boldsymbol{-sin(\delta+\alpha)} \\ \mathbf{0} & \mathbf{0} & \boldsymbol{-sin(\delta+\alpha)} & \boldsymbol{cos(\delta+\alpha)} \end{pmatrix} \cdot \begin{pmatrix} \boldsymbol{M_{00}} & \boldsymbol{M_{01}} & \boldsymbol{M_{02}} & \boldsymbol{M_{03}} \\ \boldsymbol{M_{10}} & \boldsymbol{M_{11}} & \boldsymbol{M_{12}} & \boldsymbol{M_{13}} \\ \boldsymbol{M_{20}} & \boldsymbol{M_{21}} & \boldsymbol{M_{22}} & \boldsymbol{M_{23}} \\ \boldsymbol{M_{30}} & \boldsymbol{M_{31}} & \boldsymbol{M_{32}} & \boldsymbol{M_{33}} \end{pmatrix} \cdot \mathbf{1/2} \begin{pmatrix} \mathbf{1} & \mathbf{1} & \mathbf{0} & \mathbf{0} \\ \mathbf{1} & \mathbf{1} & \mathbf{0} & \mathbf{0} \\ \mathbf{0} & \mathbf{0} & \mathbf{0} & \mathbf{0} \\ \mathbf{0} & \mathbf{0} & \mathbf{0} & \mathbf{0} \end{pmatrix} \cdot \begin{pmatrix} \mathbf{1} \\ \mathbf{0} \\ \mathbf{0} \\ \mathbf{0} \end{pmatrix} =$$

$$\mathbf{1/2} \begin{pmatrix} \boldsymbol{M_{00}+M_{01}} \\ \boldsymbol{M_{10}+M_{11}} \\ \boldsymbol{(M_{20}+M_{21})cos(\delta+\alpha)-(M_{30}+M_{31})sin(\delta+\alpha)} \\ \boldsymbol{(M_{20}+M_{21})sin(\delta+\alpha)+(M_{30}+M_{31})cos(\delta+\alpha)} \end{pmatrix} = \mathbf{1/2} \begin{pmatrix} \boldsymbol{T_0} \\ \boldsymbol{T_1} \\ \boldsymbol{T_2} \\ \boldsymbol{T_3} \end{pmatrix} \quad \text{--------(S1)}$$

Where, M_{ij} is variable value peculiar to sample, α is the residual static birefringence, and δ is the periodic phase difference between the *x* and *y* axes of the PEM operating at frequency $\omega_m/2\pi$ and is adjusted so as to act as a quarter-wave plate at an arbitrary wavelength

$$\delta = \delta^0_m \sin\omega_m t \quad \text{--------(S2)}$$

Here, δ^0_m is the peak modulator retardation. We can expand cosδ and sinδ in a Fourier series

$$\sin(\delta^0_m \sin\omega_m t) = 2J_1(\delta^0_m) \sin\omega_m t + 2J_3(\delta^0_m)\sin3\omega_m t + \ldots. \quad \text{--------(S3)}$$

$$\cos(\delta^0_m \sin\omega_m t) = J_0(\delta^0_m) + 2J_2(\delta^0_m)\cos2\omega_m t + \ldots\ldots \quad \text{--------(S4)}$$

and

$$\cos(\delta+\alpha) = 2J_2(\delta^0_m)\cos2\omega_m t \cdot \cos\alpha - 2J_1(\delta^0_m)\sin\omega_m t \cdot \sin\alpha + J_0(\delta^0_m)\cos\alpha.. \quad \text{--------(S5)}$$

$$\sin(\delta+\alpha) = 2J_1(\delta^0_m)\sin\omega_m t \cdot \cos\alpha + 2J_2(\delta^0_m)\cos2\omega_m t \cdot \sin\alpha + J_0(\delta^0_m)\sin\alpha.. \quad \text{--------(S6)}$$

$J_n(\delta^0_m)$ are Bessel functions of *n*th order. We can get the Stokes vector of the emerging light from detector as follow:

$$\mathbf{Mo} \cdot \mathbf{P_{(90)}} \cdot \mathbf{M_{(45,\,\delta)}} \cdot \mathbf{S_{(\theta)}} \cdot \mathbf{P_{(45)}} \cdot \mathbf{I_0} =$$

$$\begin{pmatrix} (P_x^2+P_y^2) & (P_x^2-P_y^2)sin2a & 0 & (P_x^2-P_y^2)cos2a \\ (P_x^2-P_y^2)sin2a & (P_x-P_y)^2cos^2 2a+2P_xP_y\,sin^2 2a & 0 & (P_x-P_y)^2cos2bsin2a \\ 0 & 0 & 2P_xP_y & 0 \\ (P_x^2-P_y^2)cos2a & (P_x-P_y)^2cos2asin2a & 0 & (P_x-P_y)^2cos^2 2a+2P_xP_y\,sin^2 2a \end{pmatrix}$$

$$\cdot 1/2\begin{pmatrix} 1 & 0 & 0 & -1 \\ 0 & 0 & 0 & 0 \\ 0 & 0 & 0 & 0 \\ -1 & 0 & 0 & 1 \end{pmatrix}\cdot 1/2\begin{pmatrix} T_0 \\ T_1 \\ T_2 \\ T_3 \end{pmatrix}$$

$$= 1/4\begin{pmatrix} (P_x^2+P_y^2)(T_0-T_3)+(P_x^2-P_y^2)cos2a(-T_0+T_3) \\ \left(P_x^2-P_y^2\right)sin2a(T_0-T_3)+(P_x^2-P_y^2)cos2asian2a(-T_0+T_3) \\ 0 \\ \left(P_x^2-P_y^2\right)cos2a(T_0-T_3)+\left[\left(P_x^2+P_y^2\right)cos^2 2a+2P_xP_y\,sin^2 2a\right](-T_0+T_3) \end{pmatrix}$$

$$= 1/4\begin{pmatrix} X_0 \\ X_1 \\ 0 \\ X_3 \end{pmatrix} \qquad \text{--------(S7)}$$

$D\cdot Mo\cdot P_{(90)}\cdot M_{(45,\delta)}\cdot S_{(\theta)}\cdot P_{(45)}\cdot I_0 =$

$$\begin{pmatrix} (P_x'^2+P_y'^2) & (P_x'^2-P_y'^2)sin2b & 0 & (P_x'^2-P_y'^2)cos2b \\ (P_x'^2-P_y'^2)sin2b & (P_x'-P_y')^2cos^2 2b+2P_x'P_y'sin^2 2b & 0 & (P_x'-P_y')^2cos2bsin2b \\ 0 & 0 & 2P_x'P_y' & 0 \\ (P_x'^2-P_y'^2)cos2b & (P_x'-P_y')^2cos2bsin2b & 0 & (P_x'-P_y')^2cos^2 2b+2P_x'P_y'sin^2 2b \end{pmatrix}\cdot$$

$$1/4\begin{pmatrix} X_0 \\ X_1 \\ 0 \\ X_3 \end{pmatrix}$$

The intensity, I_d, of the emerging light from the detector is expressed as

$$I_d = [(P'^2_x + P'^2_y) - (P'^2_x - P'^2_y)\sin 2b][(T_0 - \{T_2\sin(\delta+\alpha) + T_3\cos(\delta+\alpha)\}]$$
$$= [(P'^2_x + P'^2_y) + (P'^2_x - P'^2_y)\sin 2b]\{[(P_x^2 + P_y^2) + (P_x^2 - P_y^2)\sin 2a](M_{00} + M_{01})$$
$$- [(P_x^2 + P_y^2) + (P_x^2 - P_y^2)\sin 2a](M_{20} + M_{21})\sin(\delta+\alpha)$$
$$- [(P_x^2 + P_y^2) + (P_x^2 - P_y^2)\sin 2a](M_{30} + M_{31})\cos(\delta+\alpha)\} \qquad \text{--------(S8)}$$

By using a lock-in amplifier being tuned to $\omega/2\pi$, we can detect the LB as 50 kHz signal, and the output signal is given as

$$I(\omega) = (4G_3/\pi)\cdot[(P'^2_x + P'^2_y) - (P'^2_x - P'^2_y)\sin 2b][(P_x^2 + P_y^2) + (P_x^2 - P_y^2)\sin 2a]$$

$$\times\{-(M_{20} + M_{21})\,(2J_1(\delta^0_m)\sin\omega_m t) - (M_{30} + M_{31})\,(2J_1(\delta^0_m)\sin\omega_m t\,\sin\alpha)\}\text{-------(S9)}$$

The factors of 4/π which have been introduced into the ω response arise from the properties of the lock-in amplifier which average the absolute magnitude of the sinusoidal signals over time. G_3 is an apparatus constant related to the sensitivity of the spectrometer with the polarizer inserted. Thus, the output to a recorder, I_{out} ($=I(\omega)/I_{DC}$), is expressed as

$$I_{out} = (8G_3/\pi)\cdot P\,[-(M_{20} + M_{21}) - (M_{30} + M_{31})\sin\alpha]/P\cdot(M_{00} + M_{01})$$

$$= (8G_3/\pi)[-(M_{20} + M_{21}) - (M_{30} + M_{31})\sin\alpha]/(M_{00} + M_{01}) \qquad \text{--------(S10)}$$

Here, P, M_{00}, M_{01}, M_{20}, M_{21}, M_{30} and M_{31} are $[(P'^2_x + P'^2_y) - (P'^2_x - P'^2_y)\sin 2b]\cdot[(P^2_x + P^2_y) + (P^2_x - P^2_y)\sin 2a]$, $e^{-Ae}[1 + (LD^2 + LD'^2)/2]$, $e^{-Ae}[-(LD'\cos2\theta + LD\sin2\theta)]$, $e^{-Ae}[CD + (LD'LB - LDLB')/2]$, $e^{-Ae}[LB\cos2\theta - LB'\sin2\theta]$, $e^{-Ae}[LD'\sin2\theta - LD\cos2\theta)]$ and $e^{-Ae}[CB + (LD^2 + LB^2 - LD'^2 - LB'^2)\sin4\theta/2 + (LDLD' + LBLB')\cos4\theta]$, respectively. Where, θ is the rotation angle of the sample, Ae is the mean absorption, and LD′ and LB′ are 45° linear dichroism and birefringence, respectively. The denominator is approximated to 1 because $1 \gg |LD'\cos2\theta + LD\sin2\theta| > 1/2(LD^2 + LD'^2)$. Thus, we can approximate Eq. (S10) as

$$I_{out} = (8G_3/\pi)[-(M_{20} + M_{21}) - (M_{30} + M_{31})\sin\alpha] \qquad \text{--------(S11)}$$

Expanding M_{20}, M_{21}, M_{30} and M_{31}, the 50 kHz signal is rewritten as

$$\text{Signal}_{50kHz} = (8e^{-Ae}G_3/\pi)[CD + 1/2(LD'LB - LDLB') - LB\cos2\theta + LB'\sin2\theta$$
$$-\{LD'\cos2\theta - LD\sin2\theta - CB + 1/2(LD^2 + LB^2 - LD'^2 - LB'^2)\sin4\theta + (LDLD'+LBLB')\cos4\theta\}\sin\alpha] \qquad \text{--------(S12)}$$

We can set CD = CB = 0 for optically inactive samples having macroscopic anisotropy. Then,

$$\text{Signal}_{50kHz} = (8e^{-Ae}G_3/\pi)[\,1/2(LD'LB - LDLB') - LB\cos2\theta + LB'\sin2\theta$$
$$-\{LD'\cos2\theta - LD\sin2\theta + 1/2(LD^2 + LB^2 - LD'^2 - LB'^2)\sin4\theta + (LDLD'+LBLB')\cos4\theta\}\sin\alpha] \qquad \text{--------(S13)}$$

Here, the higher-order terms multiplied by sinα can be neglected because sinα is of the order of 10^{-3} for the PEM used in the current CCS. Thus, Eq.(S13) can be rewritten as

$$\mathrm{Signal}_{50\mathrm{kHz}} = (8e^{-Ae}G_3/\pi)[\ 1/2(LD'LB - LDLB') - LB\cos 2\theta + LB'\sin 2\theta]\text{-------(S14)}$$

Generally, LB is substantial compared with other polarization phenomena such as LD, CD and CB. Thus, 50 kHz signal obtained with an analyzer inserted can be generally regarded as LB signal.

Experimental results

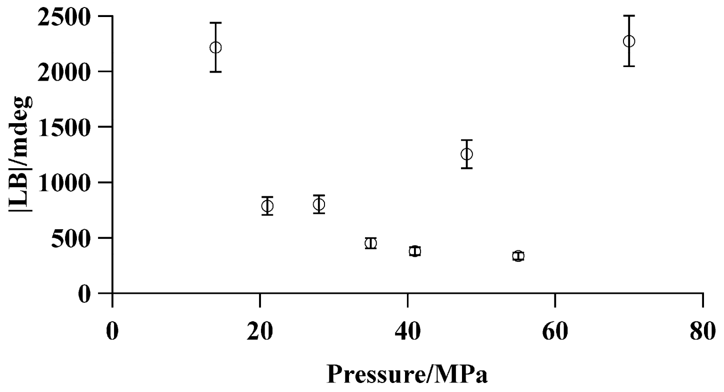

Figure S2. The absolute LB signals of KBr disks made from different pressure: 14 MPa, 21 MPa, 28 MPa, 35 MPa, 41 MPa, 48 MPa, 55 MPa and 70 MPa.

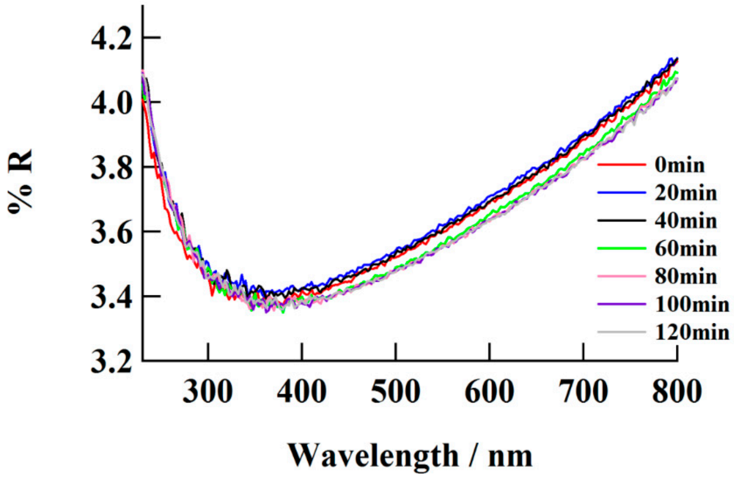

Figure S3. Time-course change of reflection spectra of KBr disk (20 min intervals).
